# SPOP suppresses pancreatic cancer progression by promoting the degradation of NANOG

**DOI:** 10.1038/s41419-019-2017-z

**Published:** 2019-10-17

**Authors:** Peng Tan, Yunke Xu, Yichao Du, Lile Wu, Bing Guo, Shiyao Huang, Jinhui Zhu, Bo Li, Fujun Lin, Lei Yao

**Affiliations:** 1 Department of Hepatobiliary Surgery, The Affiliated Hospital of Southwest Medical University, 646000 Luzhou, Sichuan China; 2 Academician (Expert) Workstation of Sichuan Province, The Affiliated Hospital of Southwest Medical University, 646000 Luzhou, Sichuan China; 3 Department of Hepatobiliary Surgery, Hospital (TCM) Affiliated to Southwest Medical University, 646000 Luzhou, Sichuan China; 4 Department of General Surgery and Laparoscopic Center, The Second Affiliated Hospital Zhejiang University School of Medicine, 310009 Hangzhou, China; 5 Renal Division, Department of Internal Medicine, Xin Hua Hospital Affiliated to Shanghai Jiao Tong University School of Medicine, 200092 Shanghai, China

**Keywords:** Pancreatic cancer, Pancreatic cancer, Tumour-suppressor proteins, Tumour-suppressor proteins

## Abstract

Speckle-type POZ domain protein (SPOP), an adaptor in the E3 ubiquitin ligase complex, recognizes substrates and promotes protein degradation via the ubiquitin-proteasome system. It appears to help regulate progression of several cancers, and we show here that it acts as a tumor suppressor in pancreatic cancer. Our analysis of patient tissues showed decreased SPOP expression, which was associated with poor prognosis. SPOP knockdown in SW1990 (in vitro/vivo) and PANC-1 (in vitro) cells led to significantly greater proliferation, migration, and invasion. Co-immunoprecipitation experiments in SW1990 cells showed that SPOP interacted with the stem-cell marker NANOG, and this interaction has recently been shown to play a critical role in regulating progression of prostate cancer. We showed that, in one patient with pancreatic cancer, the expression of a truncated form of SPOP (p.Q360*) lacking the nuclear localization signal led to nuclear accumulation of NANOG, which promoted growth and metastasis of pancreatic cancer cells. Our results suggest that SPOP suppresses progression of pancreatic cancer by promoting the ubiquitination and subsequent degradation of NANOG. These results identify the SPOP-NANOG interaction as a potential therapeutic target against pancreatic cancer.

## Introduction

Pancreatic cancer is characterized by poor prognosis and high mortality. In 2018, an estimated 458,000 new cases of pancreatic cancer and 432,000 deaths related to the disease were recorded worldwide^1^. The overall 5-year survival rate of pancreatic cancer is less than 5% with the median survival time of only 5–8 months, and the long-term survival of pancreatic cancer has not improved in the last two decades^2^. Pancreatic cancer has been associated with environmental and genetic risk factors, but how it begins and progresses remains unclear.

Ubiquitination is a post-translational modification in which mono-ubiquitin or poly-ubiquitin is added to a protein at one or several amino acid residues^3^. The length and type of ubiquitination affect the fate of the protein^4,5^. For example, lysine 48-linked poly-ubiquitination often means degradation in the ubiquitin-proteasome system (UPS), which acts as a highly selective “garbage disposal” system^6,7^. In the UPS, E3 ubiquitin ligases (E3s) are responsible for recognizing target substrates and transferring E2 ubiquitin-conjugating enzymes to substrates^8^. Many E3s contain really interesting new gene (RING) domains^9^, and several RING-containing E3s recognize substrates via an adaptor protein^8,9^. Speckle-type POZ domain protein (SPOP) is an adaptor protein in the SPOP/cullin 3 (CUL3)/ ring-box 1(RBX1) E3 complex, and it contains a meprin and tRAF homology (MATH) domain at the N-terminus, as well as a bric-a-brac/tramtrack/broad complex (BTB) domain at the C-terminus. SPOP interacts with CUL3 via the BTB domain and with specific substrates via the MATH domain^10^. Ubiquitination by the SPOP/CUL3/RBX1 complex can lead to substrate degradation, altered activity or subcellular re-localization^11,12^.

In addition to important roles in organ development^11^, SPOP-mediated ubiquitination regulates the onset and progression of various cancers. SPOP has been shown to act as a tumor suppressor in most cancers but as an oncoprotein in kidney cancer^11,12^. Recently, it has been shown to act as a suppressor in prostate cancer by limiting the stability and activity of the homeobox protein NANOG^13,14^. As a transcription factor that helps maintain stem cell pluripotency^15^ and promote self-renewal^16^ and somatic cell reprogramming^17^, NANOG can function as an oncoprotein to activate cancer stem cells and promote cancer cell cycle, immune evasion, metastasis, chemoresistance, angiogenesis, and the epithelial-mesenchymal transition (EMT)^18–20^. A meta-analysis of 2 data sets from 90 pancreatic cancer patients linked elevated NANOG expression with poor overall survival^21^. Recent studies with patient samples and cell lines indicate that SPOP-mediated degradation of NANOG helps prevent prostate cancer progression^13,14^. We are unaware of studies examining the potential role of SPOP or the SPOP-NANOG interaction in onset and progression of pancreatic cancer.

Therefore, we used patient samples and cell lines of pancreatic cancer to examine whether SPOP activity may be associated with the onset or progression of this disease and, if so, whether SPOP exerts its effects through its role in promoting the ubiquitination-dependent degradation of NANOG.

## Results

### Low expression of SPOP correlates with poor prognosis in pancreatic cancer

**Fig. 1:**
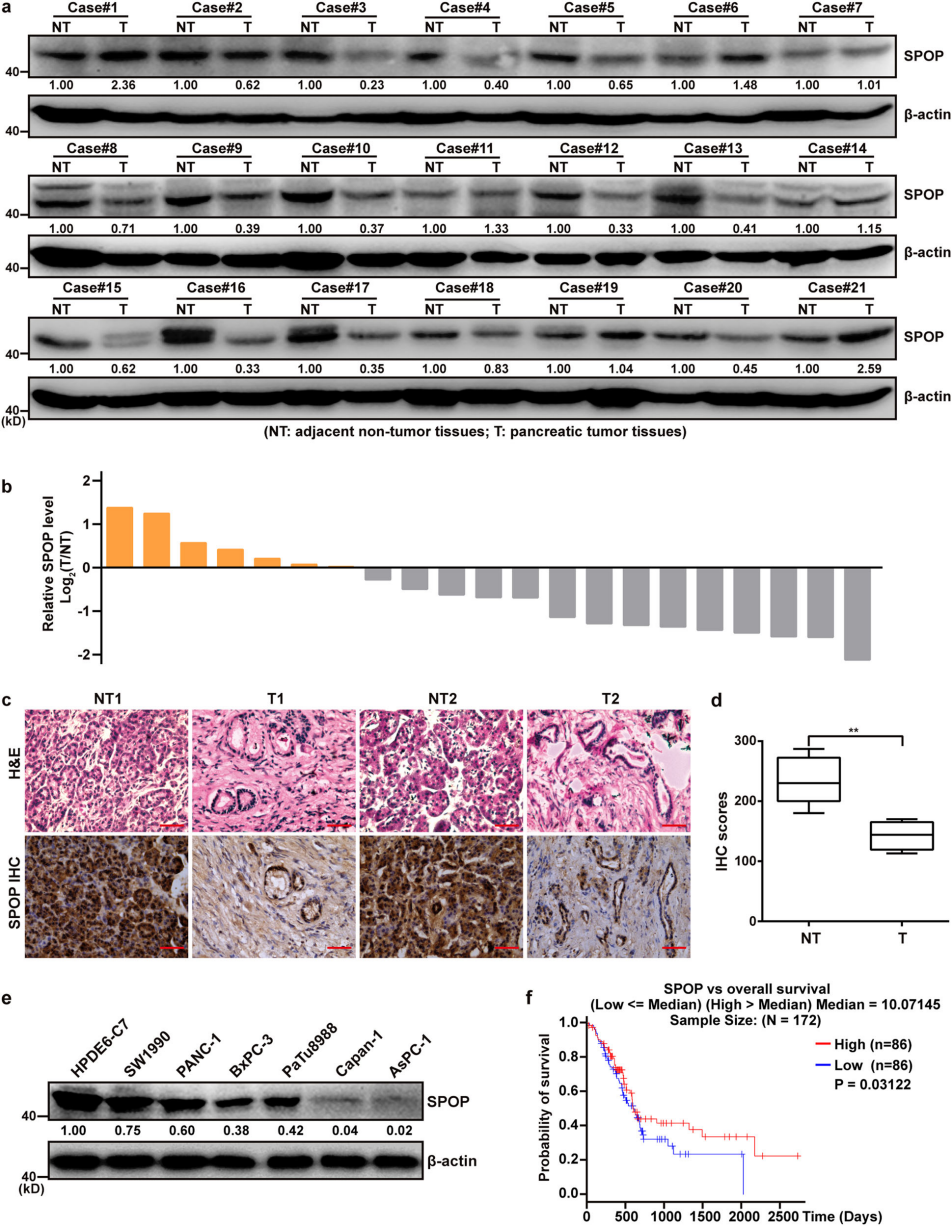
Low expression of SPOP correlates with poor prognosis in pancreatic cancer. **a** Western blot analysis of SPOP protein expression in adjacent non-tumor tissues and pancreatic tumor tissues. **b** The bar graph of SPOP protein expression in 21 pairs of pancreatic tumor tissues compared to adjacent non-tumor tissues. Data are presented as log_2_(T/NT), upregulation (orange), and downregulation (gray). **c** Representative hematoxylin-eosin (H&E) staining and immunohistochemical (IHC) analysis of SPOP in pancreatic cancer samples. Scale bars = 100 μm. **d** Statistical analysis of the average score of SPOP staining between 4 pairs of pancreatic tumor tissues and corresponding non-tumor tissues. **P < 0.01. **e** SPOP expr**e**ssion in six pancreatic cancer cell lines compared to the normal cell line HPDE6-C7, based on western blotting. **f** Kaplan–Meier survival analysis of 172 pancreatic cancer patients with high (above median, red) or low (below median, blue) SPOP expression. The gene expression data with patient survival times were obtained from the pancreatic adenocarcinoma (PAAD) dataset in The Cancer Genome Atlas (TCGA), using the Linkedomics platform (http://www.linkedomics.org)

A total of 21 pancreatic ductal adenocarcinoma tissues and adjacent non-tumor tissues were assayed for SPOP levels using western blotting (WB) and immunohistochemistry. In 14 of 21 patients (66.7%), SPOP expression was lower in tumor tissue than non-tumor tissue, it was more than 2-fold lower in 9 patients (Fig. 1a, b). Immunohistochemistry showed SPOP expression tended to be lower in tumor tissue than adjacent non-tumor tissue (Fig. 1c, d). Of six pancreatic cell lines (SW1990, PANC-1, BxPC-3, AsPC-1, Capan-1, and PaTu8988), all showed lower SPOP expression than the human normal pancreatic duct epithelial cell line (HPDE6-C7) (Fig. 1e). Kaplan–Meier survival analysis of pancreatic adenocarcinoma patients in The Cancer Genome Atlas (TCGA) showed that decreased SPOP expression was associated with worse prognosis (Fig. 1f). These results suggest that downregulation of SPOP is associated with poor prognosis in pancreatic cancer and SPOP may negatively regulate pancreatic cancer progression.

### SPOP downregulation promotes proliferation, migration, and invasion of pancreatic cancer in vitro

**Fig. 2:**
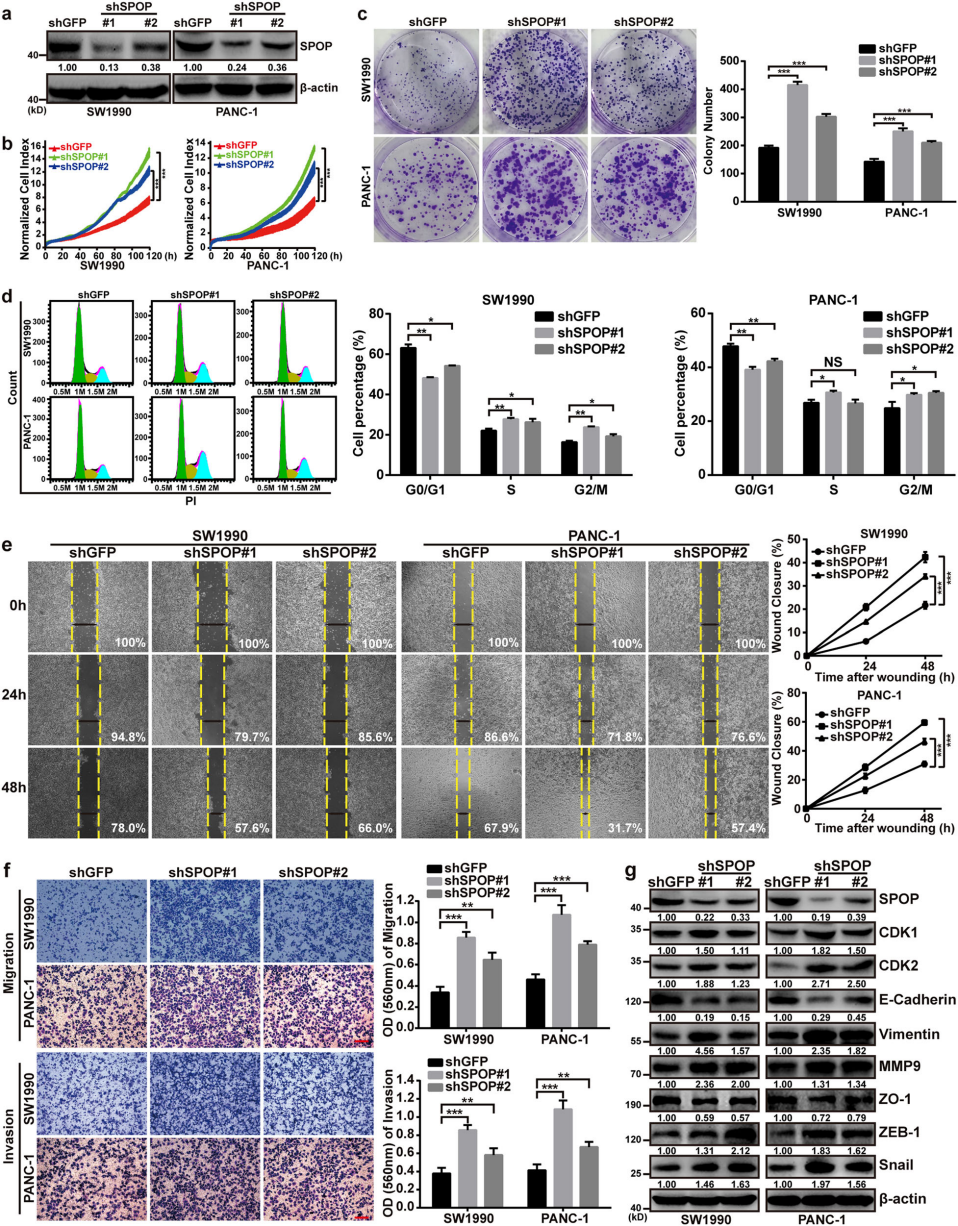
SPOP downregulation promotes proliferation, migration and invasion of pancreatic cancer in vitro. **a** SW1990 and PANC-1 cells were transfected with shSPOP#1 or shSPOP#2 sequences, with shGFP as a control. The efficiency of shRNA-mediated interference was detected using western blotting. **b** Dynamic monitoring of the proliferation of SW1990 and PANC-1 cells after SPOP knockdown using the iCELLigence RTCA analyzer. ***P < 0.001. **c** Effects of SPOP knockdown on colony formation by SW1990 and PANC-1 cells. Quantification of the stained colonies is shown in the right panel. Data are the average of three experiments (mean ± SD). ***P < 0.001. **d** Cell cycle analysis of SW1990 and PANC-1 cells after SPOP knockdown using flow cytometry. Quantification of cell percentage is presented on the right panel. NS, *P* > 0.05; *P < 0.05; **P < 0.01. **e** Cell motilities were measured through testing the wound closure after SPOP knockdown in SW1990 and PANC-1 cells. Quantification of the wound closure is shown in the right panel. Data are the average of three experiments (mean ± SD). ***P < 0.001. **f** Transwell assays were used to detect the migration and invasion abilities after SPOP knockdown in SW1990 and PANC-1 cells. Scale bar = 100 μm. Quantification of the stained colonies is shown in the below panel. Data are the average of three experiments (mean ± SD). **P < 0.01; ***P < 0.001. **g** Western blot analysis was performed to characterize the expression of some cell cycle regulatory proteins and key metastasis-related proteins in SW1990 and PANC-1 cells in which SPOP was knocked down

The pancreatic cell lines SW1990 and PANC-1 were infected with lentiviruses containing shSPOP#1 or shSPOP#2 in order to generate stable cell lines in which SPOP expression was knocked down (Fig. 2a). This knockdown significantly increased proliferation and colony formation of SW1990 and PANC-1 expressing shSPOP#1 or shSPOP#2 (Fig. 2b, c). Cell cycle experiment showed that SPOP ablation reduced the ratio of cells in the G0/G1 phase whereas increased S and G2/M phase populations compared with the control group (Fig. 2d). Meanwhile, knockdown of SPOP significantly enhanced wound healing, migration, and invasion (Fig. 2e, f). It also significantly altered expression of the cell cycle proteins cyclin-dependent kinases 1 and 2, as well as the EMT proteins E-cadherin, Vimentin, MMP9, ZO-1, ZEB-1, and Snail (Fig. 2g). These results implicate SPOP in the proliferation, invasion, and migration of pancreatic cells through SPOP’s effects on the cell cycle and EMT.

### SPOP interacts with NANOG and accelerates its poly-ubiquitination and degradation in pancreatic cancer

**Fig. 3:**
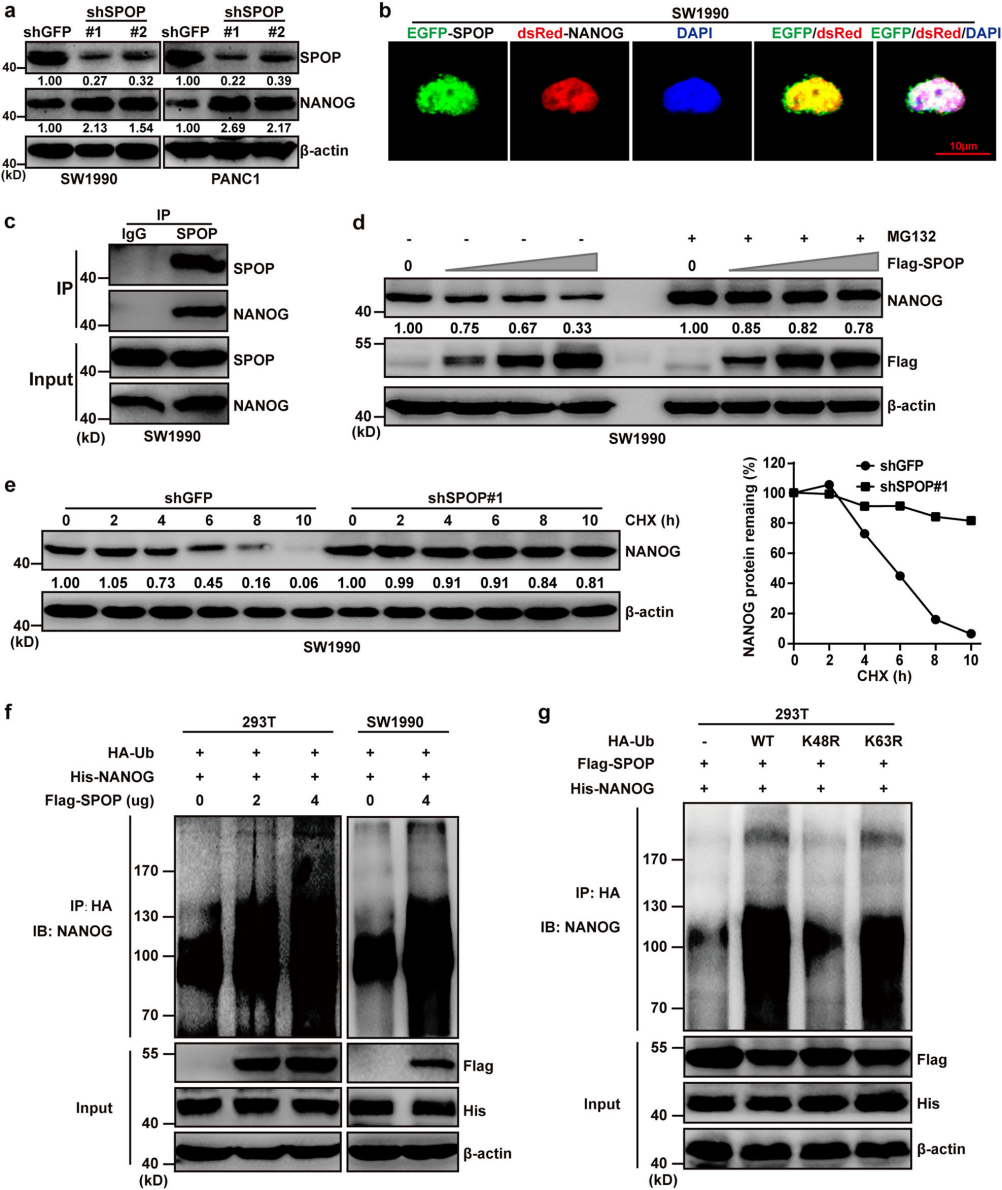
SPOP interacts with NANOG and accelerates its poly-ubiquitination and degradation in pancreatic cancer. **a** SW1990 and PANC-1 cells were transfected with shSPOP#1 or shSPOP#2 sequences, with shGFP as a control. The expression of NANOG was detected using western blotting. **b** Representative fluorescence photomicrographs display co-localization (white) of SPOP (green) and NANOG (red) in the nucleus (blue). The SW1990 cells were transfected with the indicated plasmids for 24 h. **c** Co-immunoprecipitation (Co-IP) assays demonstrated that SPOP interacted with NANOG in SW1990 cells after treatment with 20 μM MG132 for 8 h. **d** Western blot analysis of WCL derived from SW1990 cells transfected with or without Flag-SPOP plasmids as indicated, followed by treatment with DMSO (left four lanes) or 20 μM MG132 (right three lanes) for 8 h before harvesting. **e** Western blot analysis of WCL derived from SW1990 cells stably infected with the indicated lentiviral shRNAs against SPOP and treated with 100 μg/ml CHX for indicated times. Quantification of the band intensities using ImageJ software. **f** 293T and SW1990 cells were transfected with indicated plasmids for 24 h followed by 20 μM MG132 treatment for 8 h. Immunoprecipitated hemagglutinin-tagged ubiquitin (HA-Ub) was analyzed for ubiquitination using western blotting against NANOG. **g** HA-Ub-WT, HA-Ub-K48R or HA-Ub-K63R ubiquitin were cotransfected with Flag-SPOP and His-NANOG into 293T cells. Cells were treated with 20 μM MG132 for 8 h. Ubiquitination assays were performed to study the effect of Ub mutants on NANOG ubiquitination

Recent work has demonstrated that SPOP-NANOG interaction via the MATH domain and SBC degron allows SPOP to control NANOG activity and reduce its oncogenic potential in prostate cancer^13,14^. However, the SPOP-NANOG interaction in onset and progression of pancreatic cancer warranted further research. Therefore, we first measured the NANOG expression level in SPOP-knockdown cells using WB. The results showed that the expression of NANOG was significantly increased in SW1990 and PANC-1 shSPOP cell lines (Fig. 3a). EGFP-SPOP and dsRed-NANOG were co-expressed in SW1990 cells, and the result demonstrated co-localization and potential interaction of SPOP and NANOG in the nucleus (Fig. 3b). Further, co-immunoprecipitation experiment revealed that SPOP interacted with NANOG in SW1990 cells (Fig. 3c). NANOG degradation was reduced when MG132 was added to inhibit proteasome-dependent protein degradation (Fig. 3d), and when CHX was added to inhibit total protein synthesis in SW1990-shSPOP#1 cells (Fig. 3e). Furthermore, we also found that NANOG ubiquitination was increased in 293T and SW1990 cells that simultaneously overexpressed SPOP and NANOG (Fig. 3f). Ubiquitination assays also showed that SPOP controlled poly-ubiquitination of NANOG through Ub K48 (Fig. 3g). These results suggest that, as in prostate cancer, SPOP interacts with NANOG and accelerates its poly-ubiquitination and degradation in pancreatic cancer.

### SPOP insufficiency promotes pancreatic cancer cell proliferation in vivo

**Fig. 4:**
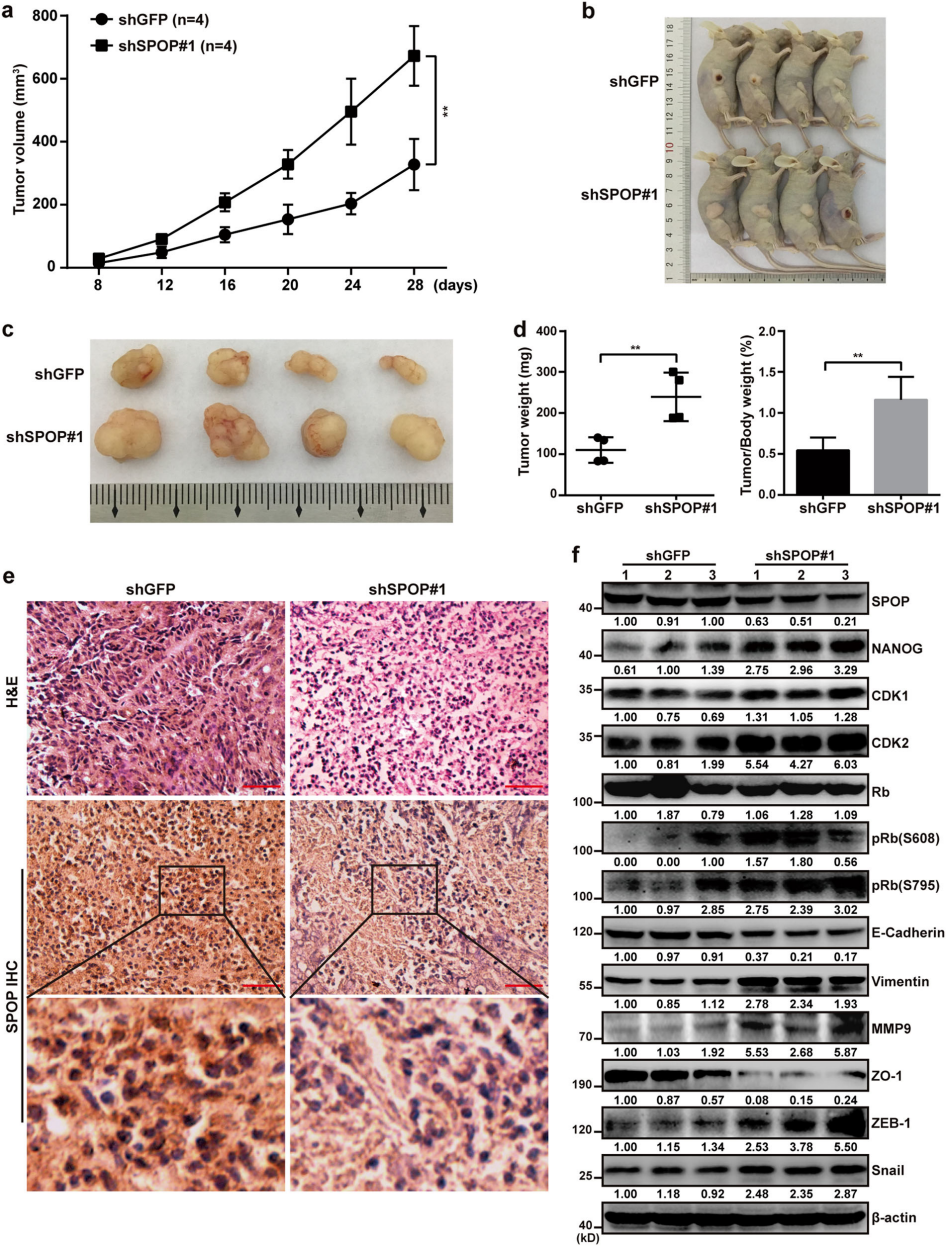
SPOP insufficiency promotes pancreatic cancer cell proliferation in vivo. **a** Mean tumor volume measured by caliper on the indicated days. Tumor volume in the curve was expressed as mean ± S.D. (*n* = 4). **P < 0.01. **b** Photographs showing the appearance of tumors in tumor-bearing nude mice. **c** Photographs of tumors excised 28 days after inoculation of stably transfected cells into nude mice. **d** Tumor weight and tumor/body weight ratios of each nude mouse at the end of 28 days. Tumor weight in the curve was expressed as mean ± S.D. (*n* = 4). **P < 0.01. **e** H&E staining and IHC analysis of SPOP in tissue samples from xenograft models. Scale bars = 100 μm. **f** Western blot analysis was performed to characterize the expression of some cell cycle regulatory proteins and key metastasis-related proteins in SW1990-Control (left three lanes) and SW1990-shSPOP groups (right three lanes)

Subcutaneous SW1990 xenografts were established in BALB/c nude mice. SW1990-shSPOP#1 tumors grew more quickly than tumors expressing normal levels of SPOP (Fig. 4a), and the larger size of shSPOP#1 tumors was confirmed by harvesting tumors at 28 days after implantation (Fig. 4b, c) and by comparing tumor weight and tumor/body weight ratio (Fig. 4d). Immunohistochemistry confirmed that SPOP was expressed at lower levels in SW1990-shSPOP#1 tumors than in SW1990-shGFP tumors in vivo (Fig. 4e). SPOP insufficiency led to upregulation of NANOG, CDK1, CDK2, pRb (S608 and S795), Vimentin, MMP9, ZEB-1, and Snail, and downregulation of E-cadherin and ZO-1 (Fig. 4f). These results further support that SPOP insufficiency promotes pancreatic cancer cell proliferation by deregulating the cell cycle and EMT.

### SPOP controls pancreatic cancer phenotype partly through NANOG

**Fig. 5:**
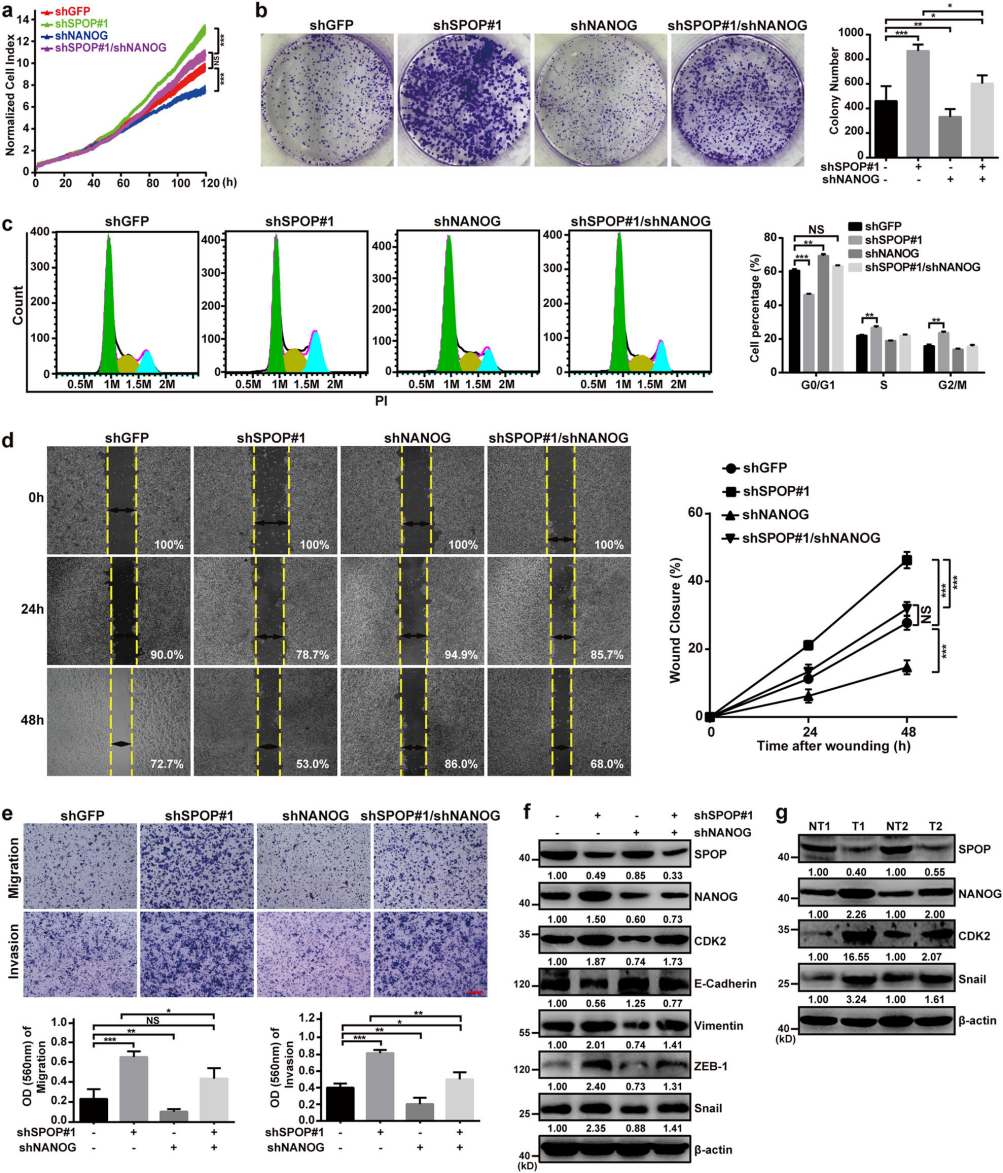
SPOP controls pancreatic cancer phenotype partly through NANOG. **a** Dynamic monitoring of the proliferation of SW1990 cells after NANOG downregulation in cells with SPOP knockdown using the iCELLigence RTCA Analyzer. NS, *P* > 0.05; ***P < 0.001. **b** The effects of NANOG down-regulation on the colony formation by SW1990 cells with SPOP knockdown. Quantification of the stained colonies is shown in the right panel. Data are the average of three experiments (mean ± SD). *P < 0.05; **P < 0.01; ***P < 0.001. **c** Cell cycle analysis of using flow cytometry after NANOG downregulation in SW1990 cells in which SPOP was knocked down. Quantification of cell percentage is presented on the right panel. NS, *P* > 0.05; **P < 0.01; ***P < 0.001. **d** Cell motilities were measured through testing the wound closure after NANOG downregulation in SW1990 cells in which SPOP was knocked down. Quantification of the wound closure is shown in the right panel. Data are the average of three experiments (mean ± SD). NS, *P* > 0.05; ***P < 0.001. **e** Transwell assays were used to detect the migration and invasion abilities after NANOG downregulation in SW1990 cells in which SPOP was knocked down. Scale bar = 100 μm. Quantification of the stained colonies is shown in the below panel. Data are the average of three experiments (mean ± SD). NS, *P* > 0.05; *P < 0.05; **P < 0.01; ***P < 0.001. **f** Western blot analysis of WCL derived from SW1990 infected with the indicated lentiviral shRNAs against SPOP and NANOG, and subjected to puromycin selection for 72 h before harvesting. **g** Representative western blot showing SPOP, NANOG, CDK2, and Snail expression in pancreatic cancer samples

To confirm whether SPOP depends at least in part on NANOG to influence pancreatic cancer cell behavior, we constructed a SW1990-shSPOP#1/shNANOG cell line expressing shRNAs targeting SPOP and NANOG. We compared the proliferation, colony formation, cell cycle, wound healing, migration, and invasion of these cells with stable cell lines expressing shRNAs against only SPOP or NANOG (Fig. 5a–e). The results suggest that NANOG acts as an oncoprotein in pancreatic cancer, and reducing its expression weakens the cancer phenotype. The oncogenic effects of knocking down SPOP with shSPOP#1 were counteracted by knocking down NANOG. Some of the cell cycle and EMT proteins altered by SPOP knockdown were restored to normal levels by simultaneously knocking down NANOG (Fig. 5f). Knocking down NANOG did not, by itself, substantially alter the expression of SPOP (Fig. 5f). These results suggest that NANOG mediates most, but not all, of the effects of SPOP on the cell cycle and EMT progression. Consistent with this idea, we confirmed that the components of the SPOP/NANOG/CDK2 and Snail regulatory pathways are present in pancreatic tissue from patients (Fig. 5g).

### The pancreatic cancer-associated SPOP mutation p.Q360* weakens the protein’s tumor suppression ability

**Fig. 6:**
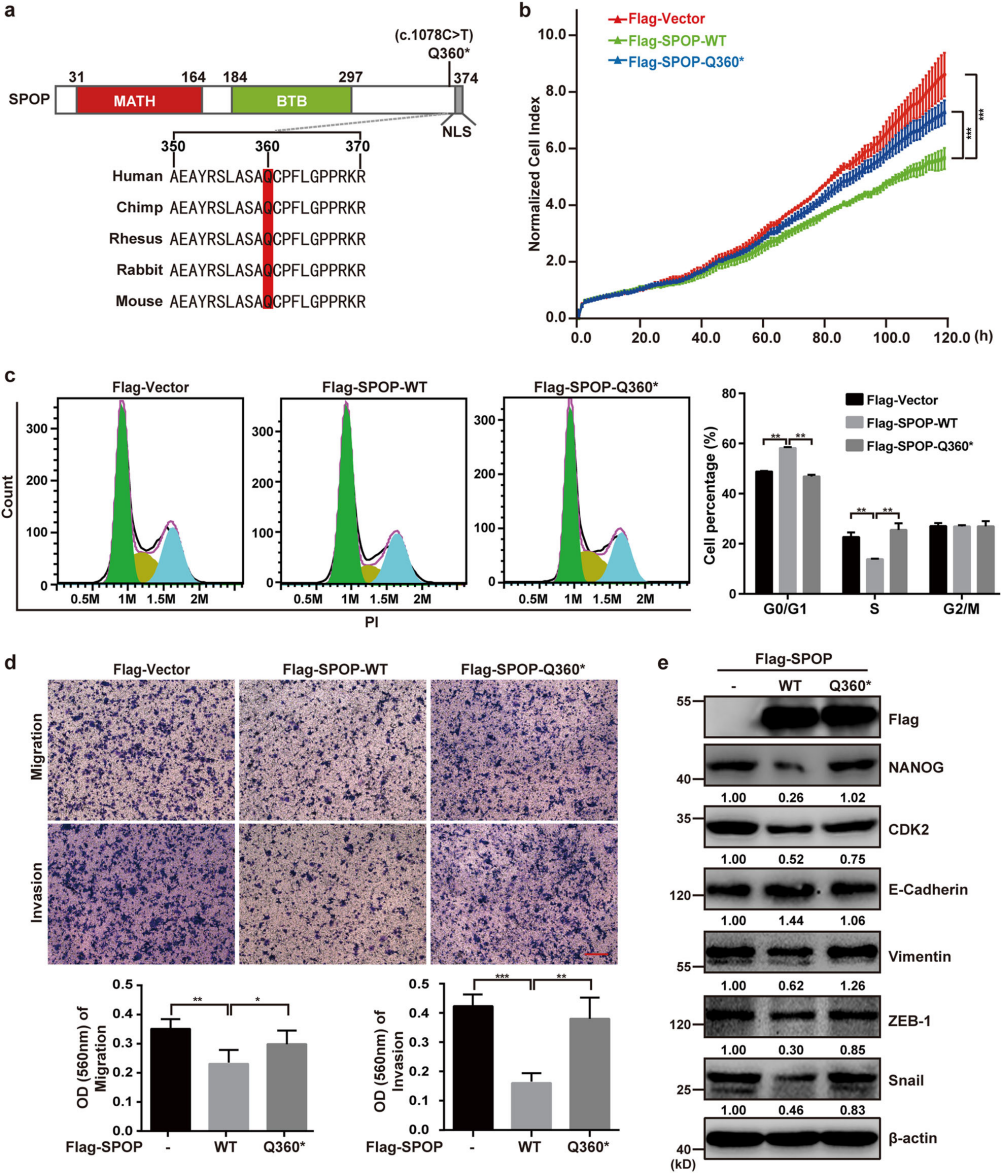
The pancreatic cancer-associated SPOP mutation p.Q360* weakens the protein’s tumor suppression ability. **a** Graphical representation of mutations of SPOP in pancreatic cancer. One “stop” mutation in SPOP was identified in the Genomic Data Commons (GDC) data sets (https://portal.gdc.cancer.gov/). Multispecies conservation of the mutated sites is shown below the mutation. NLS nuclear localization sequence. **b** Dynamic monitoring of the proliferation of SW1990 cells after transfection with Flag-SPOP-WT or Flag-SPOP-Q360* plasmids as indicated using the iCELLigence RTCA analyzer. ***P < 0.001. **c** Cell cycle analysis of SW1990 cells transfected with indicated plasmids using flow cytometry. Quantification of cell percentage is presented on the right panel. **P < 0.01. **d** Transwell assays were used to detect the migration and invasion abilities after transfection with Flag-SPOP-WT or Flag-SPOP-Q360* plasmids as indicated in SW1990 cells. Scale bar = 100 μm. Quantification of the stained colonies is shown in the below panel. Data are the average of three experiments (mean ± SD). *P < 0.05; **P < 0.01; ***P < 0.001. **e** Western blot analysis of WCL derived from SW1990 cells transfected with indicated plasmids

Data from patients with pancreatic adenocarcinoma in The Cancer Genome Atlas (TCGA) show a SPOP missense mutation (c.C1078T) that converts a glutamine codon (CAG) to a stop codon (TAG). The position Q360 site is highly conserved among mammals (Fig. 6a). Transient transfection of SW1990 cells with plasmids expressing wild-type SPOP (SPOP-WT) or SPOP-Q360* showed that the mutation led to higher cell proliferation, cell cycle progression, cell migration, and cell invasion (Fig. 6b–d). Similarly, overexpressing SPOP-Q360* did not change the levels of several downstream target proteins as much as overexpressing SPOP-WT (Fig. 6e). These results suggest that the Q360 site of SPOP may play a vital role in SPOP-mediated suppression of proliferation and metastasis of pancreatic cancer cells.

### The patient-derived Q360* mutation disrupts nuclear localization of SPOP and impairs the SPOP-mediated poly-ubiquitination and degradation of NANOG

**Fig. 7:**
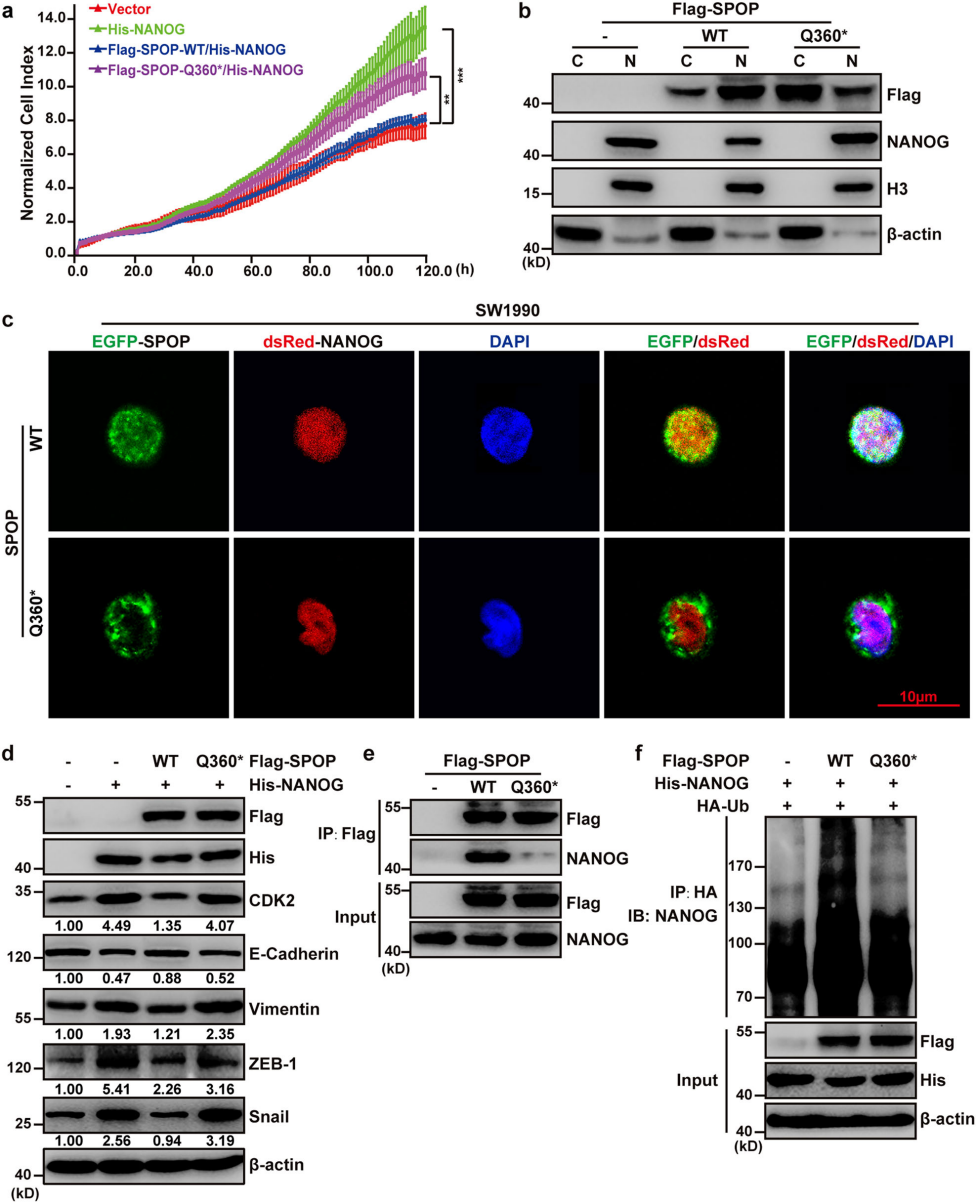
The patient-derived Q360* mutation disrupts nuclear localization of SPOP and impairs the SPOP-mediated poly-ubiquitination and degradation of NANOG. **a** Dynamic monitoring of the proliferation of SW1990 cells after transfection with indicated plasmids using the iCELLigence RTCA analyzer. **P < 0.01; ***P < 0.001. **b** Western blot analysis of SW1990 proteins separated into nuclear (N) and cytoplasmic (C) fractions. The SW1990 cells were transfected with the indicated plasmids for 48 h and separated into nuclear and cytoplasmic fractions. Histone H3 and β-actin served as nuclear and cytoplasmic markers, respectively. **c** Representative fluorescence photomicrographs displayed co-localization (white) of SPOP (green) and NANOG (red) in the nucleus (blue). The SW1990 cells were transfected with the indicated plasmids for 24 h. **d** Western blot analysis of whole-cell lysates from SW1990 cells transfected with indicated plasmids. **e** SW1990 cells were transfected with indicated plasmids. Co-IP was performed to study NANOG-SPOP interaction after treatment with 20 μM MG132 for 8 h. **f** His-NANOG and HA-Ub were expressed in SW1990 cells with wild-type or mutant SPOP. Cells were treated with 20 μM MG132 for 8 h. Ubiquitination assays were performed to study the effect of SPOP mutants on NANOG ubiquitination

To examine how the patient-derived Q360* mutation may disrupt the tumor-suppressive role of SPOP in pancreatic cancer, we transiently upregulated expression of SPOP-WT or SPOP-Q360* in cells overexpressing NANOG. We found that the Q360* mutation weakened the antiproliferative effect of SPOP under these conditions (Fig. 7a). Since the Q360* mutation is located in the linker between the BTB domain and the nuclear localization sequence, we speculated that the mutated SPOP sequence containing the C1078T mutation encodes a truncated protein without the nuclear localization sequence (Supplementary Fig. 1a-c). Indeed, fluorescence microscopy and nuclear/cytoplasmic separation showed that SPOP-WT mainly located in cell nucleus, SPOP-Q360* mainly located in cell cytoplasm, NANOG localized predominantly in the nucleus (Fig. 7b, c). The inability of SPOP-Q360* to accumulate in the nucleus was associated with an inability to bind importin subunit alpha-6 and 7 (IPOA6 and IPOA7), a shuttle carrier between nucleus and cytoplasm (Supplementary Fig. 2). SPOP-Q360* repressed levels of NANOG and other downstream proteins to a much weaker extent than SPOP-WT (Fig. 7d), and these results were associated with impaired interaction between SPOP and NANOG, as well as impaired ubiquitination of NANOG (Fig. 7e, f). These results suggest that the Q360* mutation alters SPOP localization and allows hyperaccumulation of NANOG in the nucleus, where it can promote cell proliferation.

## Discussion

Here, we provide evidence that SPOP acts as a tumor suppressor protein in pancreatic cancer. It was downregulated in most pancreatic cancer patients in our study, and low SPOP expression correlated with poor prognosis. Knocking down SPOP in the pancreatic cancer cell lines SW1990 and PANC-1 strongly promoted proliferation, migration, and invasion, and these oncogenic effects were associated with upregulation of several proteins that drive the cell cycle and EMT. These oncogenic effects of SPOP were associated with reduced ubiquitination and degradation of NANOG, and the patient-derived SPOP mutation Q360* disrupted nuclear localization of SPOP, allowing hyperaccumulation of NANOG in the nucleus, where it drove proliferation and metastasis.

Recent work has shown that SPOP helps suppress prostate cancer progression by limiting the stability and therefore activity of NANOG^13,14^. SPOP promotes the ubiquitination and subsequent UPS-dependent degradation of NANOG, which depends on NANOG-SPOP interactions involving the MATH domain of SPOP and the SBC degron (nonpolar AA-polar AA-Serine-Serine/Threonine-Serine/Threonine, φ-π-S-S/T-S/T) of NANOG (^66^PDSST^70^). Here we provide evidence that, SPOP-NANOG interaction allows SPOP to control NANOG activity and reduce its oncogenic potential in pancreatic cancer. In recently published studies, SPOP substrates such as BRD2/3/4^22^, FASN^23^, ATF2^24^, ERG^25^, AR^26^, PTEN^27^, etc., all contain the SBC degron (φ-π-S-S/T-S/T), and SPOP recognizes SBC degron to promote its multimeric ubiquitination. This provides strong evidence for further searching for new SPOP substrates in pancreatic cancer.

Similar to our results with pancreatic cancer, several studies have found SPOP to be downregulated in primary tumors of non-small cell lung cancer^28^, liver cancer^29^, osteosarcoma^30^, colorectal cancer^31^, and gastric cancer^32^, and this downregulation was associated with poor prognosis. SPOP insufficiency may result in protein dysfunction and can no longer inhibit oncoproteins such as c-myc^33^ and glioma-associated oncogene homolog 2 (Gli2)^32^, or repress activation of the PI3K/AKT/NF-κB signaling pathway^30^ or the Hedgehog signaling pathway^28^. In our patients with pancreatic cancer, SPOP insufficiency led to higher expression of CDK2 and the EMT-related protein Snail.

SPOP mutations have been identified in prostate cancer^34^, endometrial cancer^35,36^, colorectal cancer^37^, acute lymphoblastic leukemias^38^, lung cancer^39^, and thyroid cancer^40^. In fact, one study^34^ found SPOP to be the gene mutated in the greatest proportion of prostate cancer patients (13%), with mutations occurring mainly in the MATH domain, particularly Y87, F102, W131, and F133. Prostate cancer-associated mutations of SPOP impair the interactions of SPOP with androgen receptor (AR) and with bromodomain and extraterminal domain (BET) proteins, reducing their degradation and potentially increasing resistance to anti-androgen therapy and BET inhibitors^22,26^. A different set of SPOP mutations in the MATH domain has been associated with endometrial cancer^34,35^, which weaken the ubiquitination and degradation of estrogen receptor-α (ER-α)^41^.

With TCGA data mining, we found an SPOP mutation (p.Q360*) identified in one patient with pancreatic cancer. Unlike mutations in prostate cancer, which mainly occur on the amino acid residues located on the surface of the substrate-binding pocket of the MATH domain, the Q360* mutation is located in the linker between the BTB domain and the nuclear localization sequence. The Q360* mutation terminates the protein early, and our work shows that the coding region leads to a transcript that is not degraded through nonsense-mediated mRNA decay but instead is expressed the truncated protein. The Q360* SPOP mutation re-localizes the protein from the nucleus to the cytoplasm in pancreatic cancer, which is different from mutations found in prostate cancer. We speculate that the Q360* SPOP mutation lacks a nuclear localization sequence and can no longer interact with importin subunit alpha-6 and 7, like the previously reported SPOP-ΔNLS construct^42^.

Our results identify the SPOP-NANOG interaction as a potential therapeutic target in pancreatic cancer. By extension, proteins whose expression is substantially altered by SPOP insufficiency may be suitable targets. Future studies should carefully examine these altered protein levels for therapeutic implications. Cancer-associated mutations of SPOP play diverse roles based on the substrate and context: prostate cancer-associated SPOP mutations reduce degradation of BET proteins and may increase resistance to BET inhibitors^22,26^, whereas endometrial cancer-associated SPOP mutants increase degradation of BET proteins and may sensitize cells to BET inhibitors^43^. The implications of SPOP insufficiency on downstream protein expression in pancreatic cancer should be understood comprehensively in order to optimize the development of therapeutic strategies.

We showed that the low SPOP expression and the SPOP mutant lead to decreased repression of NANOG, which can then promote pancreatic cancer cell proliferation, migration and invasion. Our findings also add SPOP mutation (p.Q360*) pancreatic cancer to the list of kidney cancer in which cytoplasmic SPOP is associated with progression^27,44^. At least one study of kidney cancer has demonstrated the possibility of specifically inhibiting cytoplasmic SPOP while allowing nuclear SPOP to function normally^45^, which may be relevant for design of novel drugs against SPOP mutation (p.Q360*) pancreatic cancer. It may also be fruitful to develop NANOG-specific inhibitors.

## Materials and methods

### Ethics statement

Our researchers complied with the International Ethical Guidelines for Biomedical Research Involving Human Subjects (CIOMS). After being informed of the aims and procedures in this study, which was approved by the Ethics Committee of the Affiliated Hospital of Southwest Medical University (Luzhou, China), all participants signed informed consents. All animal experiments were in compliance with the Guide for the Care and Use of Laboratory Animals from the US National Institutes of Health and were approved by the Ethics Committee of the Southwest Medical University.

### Cell lines, drugs, and antibodies

Cancer lines SW1990, PANC-1, BxPC-3, and AsPC-1 were purchased from the Cell Bank of the Type Culture Collection of the Chinese Academy of Sciences (Shanghai, China). Other cell lines 293T, HPDE6-C7, Capan-1 and PaTu8988 were a gift from Dr. Qingqing Li (East Hospital of Tongji University, Shanghai, China). All cell lines were cultured in media recommended by the American Type Culture Collection; media were supplemented with 10% fetal bovine serum (Catalog No. ZQ500-A, Shanghai Zhong Qiao Xin Zhou Biotechnology) and 1% penicillin and streptomycin (Catalog No. V900929, Sigma-Aldrich, Shanghai, China) at 37 °C in a humidified incubator with an atmosphere of 5% CO_2_. Cyclohexane (CHX, C7698) was purchased from Sigma-Aldrich, and the protease inhibitor MG132 was purchased from Aladdin (Shanghai, China; Catalog No. M126521).

Primary antibodies against the following targets were purchased from Proteintech (Wuhan, China): FLAG tag (Catalog Nos. 66008-3-Ig or 20543-1-AP), 6*His tag (66005-1-Ig), HA tag (51064-2-AP), SPOP (16750-1-AP), and β-actin (66009-1-Ig). Additional primary antibodies against the following targets were purchased from Cell Signaling Technology (Shanghai, China): cyclin-dependent kinase 1 (CDK1; Catalog No. 9116S), CDK2 (2546S), Rb (9309S), pRb-Ser608 (8147S), pRb-Ser795 (9301S), E-cadherin (14472S), vimentin (5741S), matrix metalloprotease-9 (MMP9; 13667S), ZO-1 (8193S), ZEB-1 (3396S), and Snail (3879S). Primary antibody against NANOG was purchased from Servicebio (Wuhan, China; Catalog No. GB11331). The following secondary antibodies conjugated to horseradish peroxidase were purchased from Proteintech: Affinipure goat anti-mouse IgG (Catalog No. SA00001-1) and Affinipure goat anti-rabbit IgG (SA00001-2).

### Patients and specimens

Cancer tissues and matched normal adjacent tissues were obtained from 21 patients undergoing surgery for pancreatic cancer in the Department of Hepatobiliary Surgery at the Affiliated Hospital of Southwest Medical University. None of the patients had received adjuvant therapy. Fresh tissue was cut into 1-mm^3^ blocks, immediately washed in cold saline, rapidly frozen in liquid nitrogen and stored at −80 °C. Final diagnosis was confirmed by two experts based on the biopsy specimens.

### Hematoxylin-eosin staining, immunohistochemistry, and scoring

Paraffin-embedded tissue specimens were sectioned at a thickness of 5 μm, deparaffinized and rehydrated. Hematoxylin-eosin staining was performed using a commercial kit (Catalog No. C0105, Beyotime, Shanghai, China) in accordance with the manufacturer’s instructions. For immunohistochemistry, antigen retrieval was performed by heating slides in the microwave for 20 min in 0.01 M citrate buffer (pH 6.0), and 3% hydrogen peroxide was added for 10 min to quench peroxidase activity. Sections were treated with normal goat serum, followed by incubation overnight with anti-SPOP antibody (1:50) at 4 °C. Sections were then rinsed with phosphate-buffered saline (PBS), incubated with secondary antibody for 1 h, stained with diaminobenzidine and counterstained with hematoxylin. Sections were dehydrated and sealed, then examined using a microscope (Olympus, Chengdu, China).

Staining intensity was semi-quantitated using the H-score method^46^. Ten fields at ×400 magnification were chosen randomly, and the cells in that field were scored as 0 (negative staining), 1 (weak staining), 2 (intermediate), or 3 (strong). For each field, the total number of cells and numbers of cells stained at each intensity were counted. The H-score was calculated following the formula: (% of cells stained at intensity category 1 × 1) + (% of cells stained at intensity category 2 × 2) + (% of cells stained at intensity category 3 × 3). H-scores varied from 0 to 300, where 300 meant that 100% of cells showed strong staining (3+). High SPOP expression was defined as an H-score ≥ 200.

### WB and Co-immunoprecipitation (Co-IP) analysis

Tissues and cells were lysed in RIPA lysis buffer (Catalog No. P0013B, Beyotime). Nuclear and cytoplasmic protein fractions were extracted from SW1990 cells using a commercial kit (Catalog No. P0027, Beyotime) according to the manufacturer’s instructions. Levels of target proteins were assayed by WB as described^24^. The following working concentrations were used for primary antibodies: anti-Flag tag, 1:1000; anti-6*His tag, 1:5000; anti-HA tag, 1:1000; anti-SPOP, 1:500; anti-GFP, 1:1000; anti-β-actin, 1:5000; anti-CDK1, 1:1000; anti-CDK2, 1:1000; anti-E-cadherin, 1:1000; anti-Vimentin, 1:1000; anti-MMP9, 1:1000; anti-ZO-1, 1:1000; anti-ZEB-1, 1:1000; anti-Snail, 1:1000; anti-NANOG, 1:500; and anti-histone H3, 1:10000. Secondary antibodies were used at 1:5000 dilution.

For co-immunoprecipitation assays, cells were lysed in Lysis Buffer for WB and IP (Catalog No. P0013, Beyotime). Lysates were incubated with indicated antibodies at 4 °C overnight on a rocker. Then lysates were immunoprecipitated with Protein A + G Agarose (Catalog No. P2012, Beyotime) for 3 h at 4 °C. Immunoprecipitates were analyzed by western blot, and levels of target proteins were normalized to those of β-actin based on quantitation using ImageJ 1.43 (W. S. Rasband, ImageJ, U. S. National Institutes of Health).

### Plasmids construction, lentiviruses production, cell transfection, and viral infection

Short hairpin RNA (shRNA) targeting human SPOP (shSPOP), human NANOG (shNANOG) and GFP (shGFP) was purchased from Sangon Biotech (Shanghai, China), and cloned into the pLKO.1 vector. The sequences of shRNA oligonucleotides against SPOP and NANOG were as follows: shSPOP#1, 5′-CCGGCACAAGGCTATCTTAGCAGCTCTCGAGAGCTGCTAAGATAGCCTTGTGTTTTTTG-3′; shSPOP#2, 5′-CCGGCACAGATCAAGGTAGTGAAATCTCGAGATTTCACTACCTTGATCTGTGTTTTTTG-3′; shNANOG, 5′-CCGGGCATCCGACTGTAAAGAATCTCTCGAGAGATTCTTTACAGTCGGATGCTTTTTTG-3′. Vector encoding pRK5-HA-Ubiquitin-WT was purchased from Zhongyuan (Beijing, China). The following cassettes were constructed by PCR and cloned into the pcDNA3.1 vector: Flag-SPOP, Flag-SPOP-N, Flag-SPOP-C, Flag-SPOP mutants (Y87N, F102C, F133L, Q360*), HA-ubiquitin mutants (K48R, K63R), His-NANOG, and His-NANOG-△SBC. In these constructs, Ubiquitin-WT corresponds to the full-length human protein; SPOP-N corresponds to an N-terminal fragment of the human protein containing amino acid residues 1–171; SPOP-C, to a C-terminal fragment of the human protein containing amino acid residues 172–374; NANOG, to the full-length human protein; and NANOG-△SBC, to human NANOG lacking amino acid residues 66–70.

For transient transfections, 293T or SW1990 cells at 70–80% confluence were transfected with plasmids using Lipo6000™ Transfection Reagent (Catalog No. C0529, Beyotime) according to the manufacturer’s instructions. At 48 h after transfection, cells were harvested for subsequent experiments.

For stable transfection of recombinant expression plasmids, lentivirus was produced by co-transfecting 293T cells with shRNA plasmid and packaging plasmids (pLP1, pLP2 and pLP/VSVG). Cell supernatants were harvested at 48 hours after plasmid transfection, then added to SW1990 cultures for 48 h. Then the infected cultures were switched to medium supplemented with puromycin (4 μg/ml) selection for one week to screen for stable cell lines, which were named SW1990-shGFP, SW1990-shSPOP#1, SW1990-shSPOP#2, SW1990-shNANOG and SW1990-shSPOP#1/shNANOG.

### Cell proliferation

Cell proliferation was analyzed with iCELLigence RTCA Analyzer (ACEA, Hangzhou, China). Cells were cultured at 5 × 10^3^/well in E-Plate L8 (Catalog No. 00300600850, ACEA). Cell index signals were obtained automatically using the RTCA Analyzer.

### Colony formation

Cells were seeded in 6-well plates at a concentration of ~1000 cells per well, cultured at 37 °C for 10 days, then fixed with 4% paraformaldehyde and stained with crystal violet. Colonies were photographed and counted.

### Cell cycle

SW1990 and PANC-1 cells were harvested and fixed in 70% ethanol at 4 °C overnight. Then the samples were incubated in a solution of 10 mg/ml of RNase and 1 mg/ml of propidium iodide (Sigma) at 37 °C for 30 min in the dark. The DNA content was determined using flow cytometry (BD Biosciences).

### Wound healing and cell migration/invasion

In the wound healing assay, cells were seeded in 6-well plates and allowed to grow to full confluence. The surface was scratched using a 10-μl pipette, cells were washed with PBS to remove debris, and cultures were incubated in serum-free DMEM for 48 h. Cells were observed and pictures were taken at indicated times.

For the migration and invasion assays, 24-well transwells (Corning, Shanghai, China) were used. Membranes were coated with Matrigel (BD Biosciences, Shanghai, China) for the invasion assay and left uncoated for the migration assay. Cells in serum-free medium were added to the upper chamber. Medium with 10% fetal bovine serum as the chemoattractant was placed in the lower chamber. At 48 h after incubation, the non-migrating cells in the upper chambers were carefully removed with a cotton swab, and migrated cells on the underside of the filter were fixed in 4% paraformaldehyde and stained with crystal violet. Cells were bleached with 33% acetic acid and detected based on absorbance at 560 nm.

### Tumor xenografts

Four-week-old male BALB/c nude mice were purchased from Chengdu Dashuo Biotechnological Company (Chengdu, China) and housed under specific pathogen-free conditions in a temperature-controlled and humidity-controlled room at the Experimental Animal Center of Southwest Medical University. Each mouse received an injection of the pancreatic cancer cells SW1990-shGFP or SW1990-shSPOP#1 (1 × 10^6^ cells in each case). Tumor size was estimated by measuring the length and width with a caliper every 4 days for 28 days, and plugging the values into the formula: size = length × (width)^2^/2. At the end of the experiment, tumors were removed and weighed.

### Protein degradation analysis

Protein degradation was analyzed using CHX chase. SW1990-shGFP and SW1990-shSPOP#1 were cultured with the protein synthesis inhibitor CHX (100 μg/ml) to inhibit de novo synthesis of NANOG. Cells were harvested at 0, 2, 4, 6, 8, and 10 h after treatment, and protein levels were assayed by Western blot.

### Ubiquitination assay

293T cells were co-transfected with the indicated plasmids and at 24 h later, treated with the protease inhibitor MG132 (20 mM) for 8 h to protect ubiquitinated proteins from degradation. Cells were then lysed in Lysis Buffer for WB and IP (Beyotime).

### Survival analysis

The prognostic potential of SPOP mRNA levels was assessed by retrieving mRNA expression and survival data from 172 patients in the pancreatic adenocarcinoma (PAAD) dataset of The Cancer Genome Atlas, and generating Kaplan-Meier curves using the Linkedomics platform (http://www.linkedomics.org)^47^.

### Statistical analysis

Experiments carried out in triplicates were analyzed with statistics and shown in the figures. Inter-group differences were assessed for significance using the two-tailed Student’s *t* test. Data were expressed as mean ± standard deviation. *P* < 0.05 was considered statistically significant.

## Supplementary information

Supplemental material
