# Supplementary material for: SPOP suppresses pancreatic cancer progression by promoting the degradation of NANOG
Source: Cell Death Dis. 2019 Oct 17;10(11):794. doi: 10.1038/s41419-019-2017-z (PMC6797744; doi:10.1038/s41419-019-2017-z)
Supplement: Supplementary file 1 — Supplemental material [file 41419_2019_2017_MOESM1_ESM.docx]

**Supplementary Information**

**
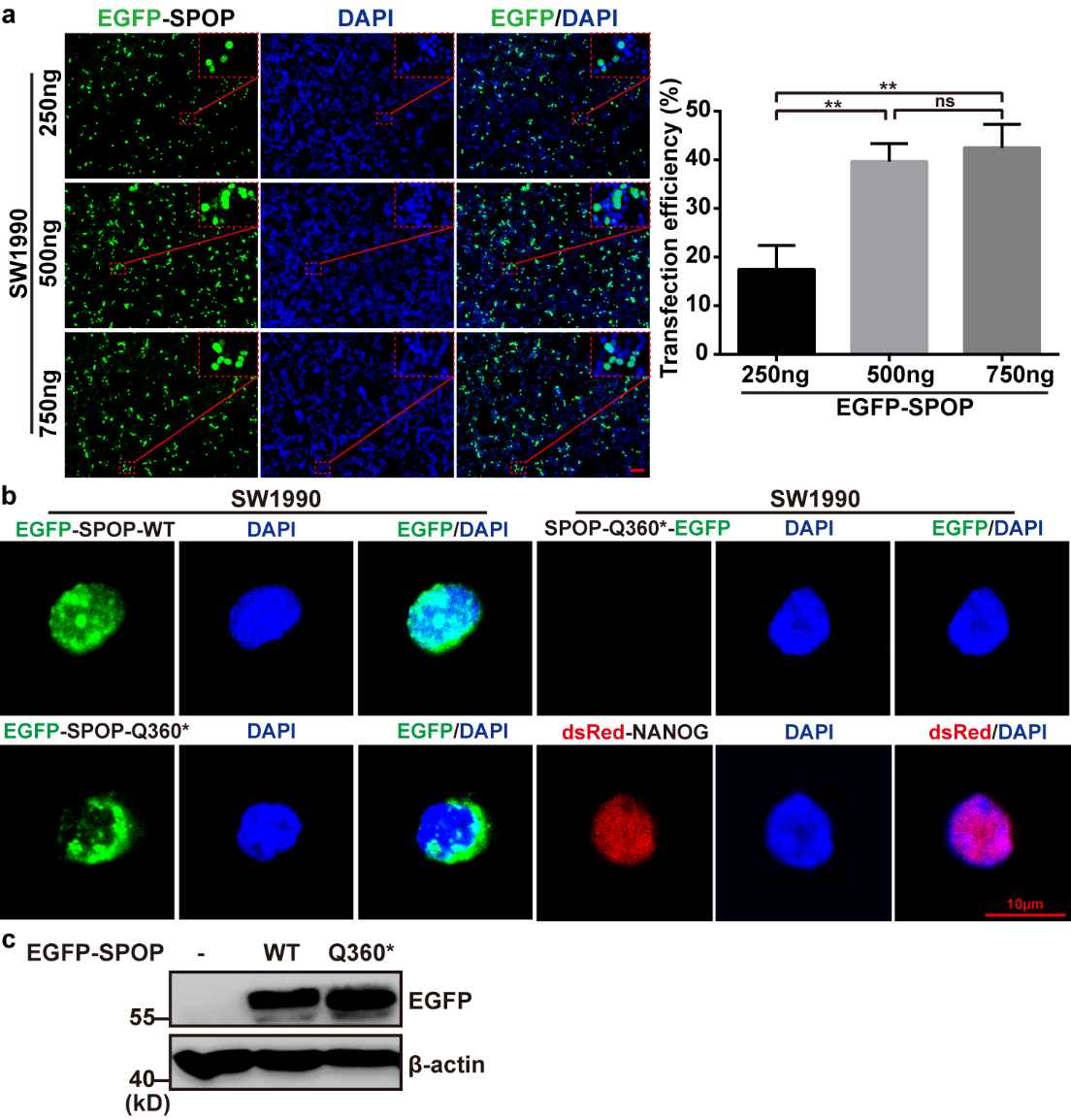
**

**Supplementary Fig. 1 Analysis of the location and expression of SPOP and NANOG in SW1990 cells.** **a** The SW1990 cells were transfected with the indicated plasmids for 48 h. Statistical analysis of the transient transfection efficiency is shown in the right panel. Scale bar = 100 μm. **b** The location of SPOP-WT (green), SPOP-Q360* (green) and NANOG (red) in SW1990 cells were determined by confocal microscopy. The SW1990 cells were transfected with the indicated plasmids for 24 h. **c** Western blot analysis of whole cell lysates from SW1990 cells transfected with EGFP-SPOP-WT or EGFP-SPOP-Q360* plasmid.


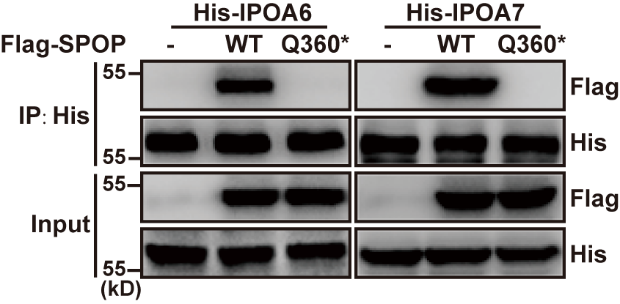


**Supplementary Fig. 2 Q360 termination mutation suppressed SPOP interaction with importin subunit alpha-6 and 7 (IPOA6 and IPOA7).** Western blot of WCL and Co-IP samples of anti-His antibody obtained from 293T cells transfected with indicated plasmids.

**
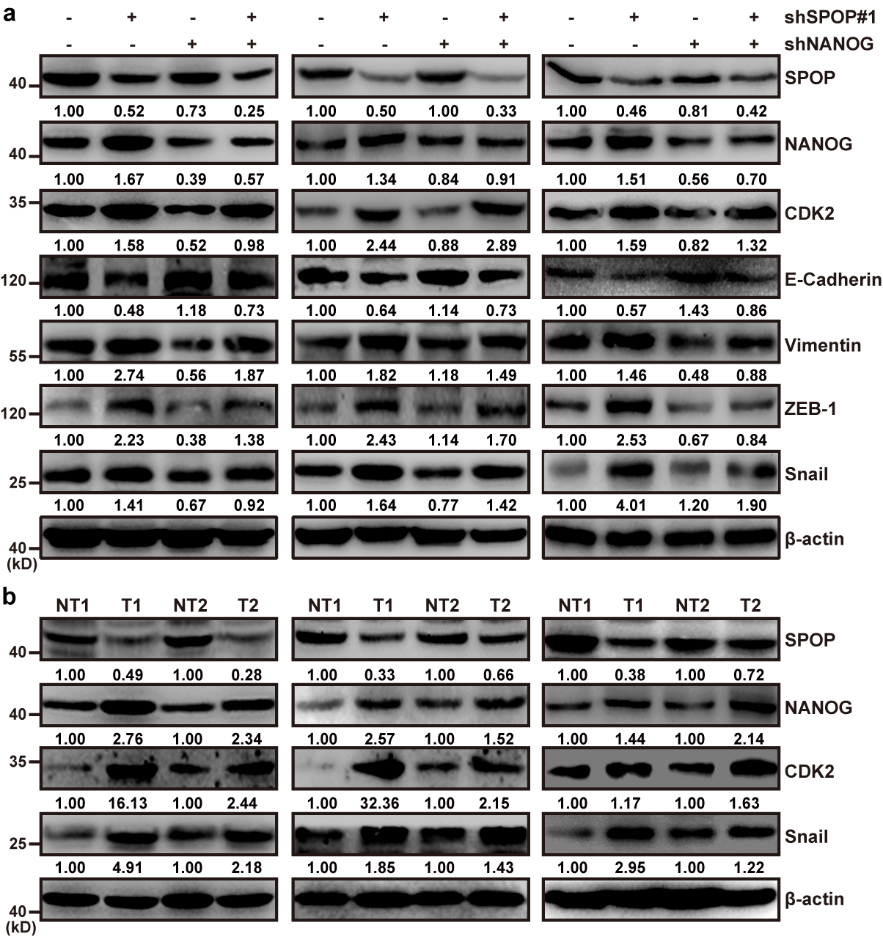
**

**Supplementary Fig. 3 The western blot experiments that were used for the quantification. a** Western blot analysis of WCL derived from SW1990 infected with the indicated lentiviral shRNAs against SPOP and NANOG, and subjected to puromycin selection for 72 h before harvesting. The experiments were repeated three times and a representative blot is shown in Fig. 5f. **b** Western blot showing SPOP, NANOG, CDK2 and Snail expression in pancreatic cancer samples. The experiments were repeated three times and a representative blot is shown in Fig. 5g.
